# Prior sick leave with mental or somatic diagnoses and being in work in ages 66–71; a Swedish cohort

**DOI:** 10.1093/eurpub/ckac130.128

**Published:** 2022-10-25

**Authors:** A Martikainen, A Svensson Alavi, K Alexanderson, K Farrants

**Affiliations:** Division of Insurance Medicine, Karolinska Institutet, Stockholm, Sweden

## Abstract

**Background:**

As longevity and retirement ages are increasing, knowledge is needed on factors hindering extended working lives. The aim of this study was to explore how sickness absence (SA) and disability pension (DP) due to mental and/or somatic diagnoses before age 65 were associated with being in paid work when aged 66-71.

**Methods:**

A 6-year prospective population-based cohort study of all 98,551 people (48% women) in Sweden who turned 65 years in 2010 (baseline year) and had been in paid work at any point when aged 60-64. Microdata from nationwide registers were used. Exposure variables were SA (spells >14 days) and/or DP in 2005-2009, and the outcome variable was paid work at any point in 2011-2016. Logistic regression was used to calculate odds ratios (OR) with 95% confidence intervals (CI) for associations between exposures and outcome, controlling for sociodemographic factors in 2010, stratified by sex.

**Results:**

Most women (56.0%) and men (66.3%) had no SA or DP when aged 60-64. Of the women, 42.7% and of the men 53.3% were in paid work after the age of 65. Those with SA due to mental diagnoses had lower OR of being in paid work (women 0.76; 95% CI: 0.69-0.84; men 0.74; 0.65-0.84). This association was weaker for SA due to somatic diagnoses (women 0.87; 0.84-0.91; men 0.92; 0.89-0.96). Having had SA due to both mental and somatic diagnoses was associated with lower OR for men (0.77; 0.65-0.91) but not women (0.98; 0.88-1.09). Full- or part-time DP had the strongest association with not being in paid work regardless of diagnosis group and sex (e.g., women mental DP 0.39; 0.34-0.45; women somatic DP 0.38; 0.35-0.41; men mental DP 0.36; 0.29-0.43; men somatic DP 0.35; 0.32-0.38).

**Conclusions:**

SA due to mental diagnoses had a stronger association with not being in paid work after age 65 than SA due to somatic diagnoses. The results highlight the importance of identifying factors that hinder older workers with mental disorders to extend their working lives.

**Key messages:**

- Many were in paid work after age 65, also among those with previous SA. However, this was less likely among those who had been on SA due to mental diagnoses compared to somatic diagnoses.
- More knowledge is needed on how older people with different types of mental and somatic morbidities can extend their working lives, what hinders and what promotes this in different European countries.

